# Abnormal Expression Pattern of the IL-2 Receptor **β**-Chain on CD4^**+**^ T Cells in ANCA-Associated Vasculitis

**DOI:** 10.1155/2014/249846

**Published:** 2014-02-09

**Authors:** Benjamin Wilde, André Hoerning, Andreas Kribben, Oliver Witzke, Sebastian Dolff

**Affiliations:** ^1^Department of Nephrology, University Hospital Essen, University Duisburg-Essen, Hufelandstraße 55, 45122 Essen, Germany; ^2^Department of Pediatrics II, Pediatric Nephrology, Gastroenterology, Endocrinology, and Transplant Medicine, Children's Hospital Essen, University Duisburg-Essen, 45122 Essen, Germany

## Abstract

*Background/Aim*. ANCA-associated vasculitis (AAV) is a small-vessel vasculitis of autoimmune origin. In addition to autoantibodies, T cells have a pivotal pathophysiological role in this disease. T-cell homeostasis and immune tolerance critically depend on IL-2 and its receptor expressed by T cells. In this study, we investigated the IL-2 receptor (IL-2r) expression on CD4^+^ T cells in AAV. *Methods*. Thirty patients with AAV and 15 age-matched healthy controls (HC) were enrolled. T cells from peripheral blood were analysed by flow cytometry for expression of the IL-2r **α**- and **β**-chain. *Results*. The IL-2r **α**-chain was overexpressed in AAV as compared to HC (36 ± 16% versus 20 ± 9%, *P* < 0.005). The IL-2r-**β**-chain expression was significantly reduced on CD25^+^ CD4^+^ T-cells and CD4^+^CD25^+^FoxP3^pos^ regulatory T-cells (Tregs; AAV versus HC: 48 ± 14% versus 62 ± 9%, *P* = 0.002 and 38 ± 18% versus 68 ± 5%, *P* = 0.002). Low **β**-chain expression in AAV was associated with relapsing disease and systemic vasculitis with renal involvement. *Conclusion*. The IL-2r expression pattern is abnormal in AAV. To our knowledge, we are the first to show that the **β**-chain expression is drastically diminished on T cells in AAV and related to a less favorable disease course. Given the indispensable function of the **β**-chain in IL-2 signaling of T cells, diminished expression may contribute to disturbed immune homeostasis in AAV.

## 1. Introduction

ANCA-associated vasculitis (AAV) is a necrotizing small-vessel vasculitis of autoimmune origin, which is characterized by the presence of antineutrophil-cytoplasmic-antibodies (ANCA) [1]. ANCA have a dominant pathogenic role in AAV and are either directed against proteinase-3 (PR3) or myeloperoxidase (MPO) [1]. Recent data suggest that, in addition to autoantibodies, T cells are pathogenic factors in AAV [2]. The isotype of the autoantibodies indicates that a T-cell dependent class switch has taken place [3]. Furthermore, T-cell infiltrates are present in organ lesions commonly observed in AAV [2, 4, 5]. In addition, granuloma formation—which is regarded as a T-cell driven process—is a histological feature of specific AAV subtypes [6]. In line with this, several phenotypical and functional T-cell abnormalities have been reported in AAV patients [2, 7–9]. T-helper cells are persistently activated which is indicated by an expansion of proinflammatory effector memory T-helper cells and upregulation of the IL-2 receptor (IL-2r) *α*-chain (CD25) [2, 10–12]. In contrast, function of anti-inflammatory T cells such as regulatory T cells (Tregs) seems to be impaired [13, 14].

IL-2 is essential for the homeostasis Tregs and effector T cells (Teff) [15, 16]. There are three different subunits of the IL-2r: *α*-chain (CD25), *β*-chain (CD122), and *γ*-chain (CD132, constitutively expressed by all lymphoid cells) [15–17]. Both the *β*-chain and the *γ*-chain have intracellular, signal transducing domains which are indispensable for proper function of the IL-2r [16]. Interestingly, CD122 is also the *β*-subunit of the IL-15 receptor and mediates signal transduction of this cytokine. Moreover, both the IL-2r *α*-chain and the *β*-chain have IL-2 binding domains. The IL-2r exists in two forms: as a heterodimer consisting of the *β*-chain and the *γ*-chain forming the low-affinity IL-2r and secondly as a heterotrimer consisting of all three chains forming the high-affinity IL-2r [16]. Teff usually express the low-affinity IL-2r and upregulate the *α*-chain to form the high-affinity receptor only transiently upon activation [16–18]. Tregs, which control Teff responses, usually express the high-affinity IL-2 receptor [16–18].

In contrast to Teff, the survival and functionality of Tregs—and thus immune tolerance—is critically dependent on IL-2 [15, 19–21]. The lack of a functional IL-2r leads to detrimental autoimmunity due to breakdown of immune tolerance [16]. In murine models, the knockdown of the IL-2r *β*-chain results in an expansion of activated Teff and reduced development of functional Tregs followed by lethal, autoimmune organ inflammation [16, 22]. This underscores the importance of the IL-2r and especially of the IL-2r *β*-chain for T-cell homeostasis and immune tolerance.

In AAV, T-cell homeostasis is persistently disturbed and regulatory mechanisms seem to be impaired [2]. Considering the important role of the IL-2r and in light of the fact that the *β*-chain CD122 has never been studied in AAV, it was the aim of this study to investigate the IL-2r expression on CD4^**+**^ T cells in AAV and its implications for disease pathogenesis.

## 2. Material and Methods

### 2.1. Patient Cohort

Thirty-one consecutive patients with AAV visiting the outpatient clinic of the Department of Nephrology were enrolled (mean age 58 ± 14 years; 17 males, and 14 females). Four of these 31 patients were sampled during active disease. Three of the four were sampled again during follow-up in remission. Three of the four patients with active AAV were untreated at the time of sampling; one had already received one cycle of intravenous cyclophosphamide. The four active patients presented with a Birmingham vasculitis activity score (BVAS) of 16, 16, 21, and 6, respectively. Twenty-seven patients were in remission at the time of sampling and nine were sampled a second time during follow-up. Remission was defined according to Hellmich et al. as the complete absence of active clinical disease reflected by a BVAS of 0 [23]. PR3-ANCA was detectable in 29 patients at the time of diagnosis; two patients had ANCA with specificity for MPO at the time of diagnosis. Sixteen of the patients sampled during remission were treated with mycophenolate mofetil (MMF) at the time of sampling; seven received azathioprine (AZA), four were treated with cyclophosphamide (CYC), one patient was treated with cotrimoxazole, and another one with methotrexate (MTX). Low-dose steroids <10 mg/day were administered to 21 patients with quiescent disease in addition to MMF, AZA, or CYC. Steroids alone without MMF, AZA, or CYC were given to one patient. The diagnosis of AAV was made in accordance with the criteria of the American College of Rheumatology and Chapel Hill consensus [24–26]. According to the definitions published by Hellmich et al., AAV was classified as localized disease without renal involvement in eight patients, whereas the remaining patients had systemic AAV with biopsy-proven renal involvement (Table 1) [23]. The mean disease duration at the time of sampling was 99 ± 80 months. Clinical data was obtained retrospectively based on patient file records. According to Hellmich et al., a relapse was defined as reactivation of disease attributable to active inflammation requiring intensified prednisone and/or therapy with AZA, CYC, MTX, or MMF. Applying this definition, 26 relapses in 14 patients were found; the median Birmingham vasculitis activity score was 10.

Fifteen age-matched healthy controls (HC, mean age 53 ± 7 years; 6 males and 9 females) with no history of chronic infections, cancer, or autoimmune diseases were enrolled as control cohort. Patients with IgA nephropathy (*n* = 18, mean glomerular filtration rate (GFR) = 45 ± 14 mL/min) and six patients with unilateral nephrectomy (*n* = 5 due to living-related kidney donation, and *n* = 1 due to renal cell cancer 10 years ago; mean GFR = 45 ± 8 mL/min) served as additional control cohorts. Informed consent and approval by the local ethics committee of the University Hospital Essen were obtained.

### 2.2. Flow Cytometry: Surface and Intracellular Staining

**Figure 1:**
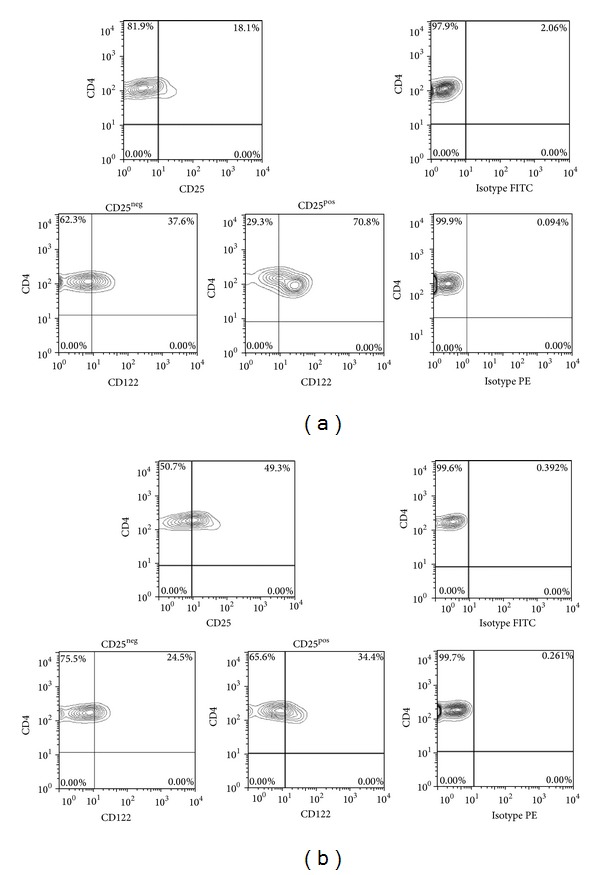
Gating strategy to determine CD122 expression on CD25^pos^ CD4^+^ T cells and CD25^neg^ CD4^+^ T cells. (a) Representative raw data of a healthy control (HC). Top panels are gated on CD4^**+**^ T cells, and bottom panels are gated on either CD25^neg^ or CD25^pos^ CD4^**+**^ T cells. (b) Representative raw data of an AAV patient. Top panels are gated on CD4^**+**^ T cells, and bottom panels are gated on either CD25^neg^ or CD25^pos^ CD4^**+**^ T cells.

**Figure 2:**
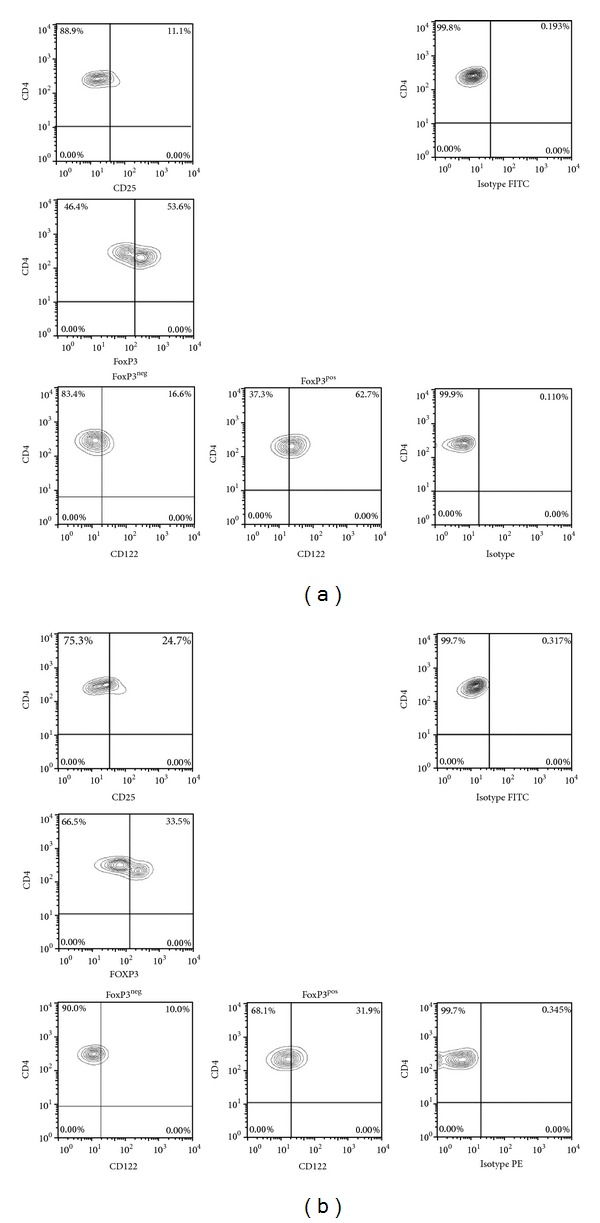
Gating strategy to determine CD122 expression on CD4^+^CD25^pos^FoxP3^neg^ T-helper cells and CD4^+^CD25^pos^FoxP3^pos^ regulatory T cells. (a) Representative raw data of a healthy control (HC). Top panels are gated on CD4^**+**^ T cells, the middle panel is gated on CD25^pos^ CD4^**+**^ T cells, and the bottom panels are gated on either FoxP3^pos^CD25^pos^ CD4^**+**^ T cells or FoxP3^neg^CD25^pos^ CD4^**+**^ T cells. (b) Representative raw data of an AAV patient. Top panels are gated on CD4^**+**^ T cells, the middle panel is gated on CD25^pos^ CD4^**+**^ T cells, and the bottom panels are gated on either FoxP3^pos^CD25^pos^ CD4^**+**^ T cells or FoxP3^neg^CD25^pos^ CD4^**+**^ T cells.

Expression levels of the receptors were measured by multicolour surface staining on unstimulated lymphocytes from whole blood. Phycoerythrin (PE), fluorescein isothiocyanate (FITC), peridin chlorophyll protein (PerCP), and allophycocyanin- (APC-) labeled antibodies with different specificity were used: CD4 (mouse IgG1, PerCP), CD25 (mouse IgG1, FITC), and CD122 (mouse IgG1, PE). Appropriate isotype controls (Becton Dickinson, Heidelberg) were used. Peripheral whole blood was stained with labeled monoclonal antibodies for 20 min at room temperature followed by red blood cell lysis. Intracellular staining for FoxP3 (clone 259D/C7, APC, Becton Dickinson) was performed on ficoll separated PBMC of patients and HC by using a fixation/permeabilization kit according to the manufacturer's instructions. A surface staining was performed with anti-CD4, anti-CD25, anti-CD122, or appropriate isotype controls followed by fixation and permeabilization (FoxP3 Fixation/permeabilization kit, Becton Dickinson, Heidelberg, Germany). Afterwards, intracellular staining with anti-FoxP3 was performed. Measurements were performed with a fluorescence activated cell sorter (FACS) Calibur from Becton Dickinson. The FACS data was analyzed by using the software Flow Jo Version 7.6.5 (Treestar Inc., Ashland, USA). Regulatory T cells were defined as CD4^**+**^CD25^**+**^FoxP3^pos^ and activated T-helper cells were defined as CD4^+^CD25^+^FoxP3^neg^. The gating strategy for FACS analysis of CD122 expression on CD25^**+**^CD4^**+**^ and CD25^neg^CD4^**+**^ T cells is given in Figures 1(a) and 1(b). The gating strategy to determine expression of CD122 on CD4^+^CD25^+^FoxP3^+^ Tregs and CD4^+^CD25^+^FoxP3^neg^ activated T-helper cells is given in Figures 2(a) and 2(b).

### 2.3. Statistics

All values are expressed as mean ± standard deviation. Significance for the differences between groups was determined using the Mann-Whitney *U* test. Matched pair analysis was performed using Wilcoxon signed-rank test. Spearman's rank correlation was applied for detecting correlations between different study parameters.

## 3. Results

### 3.1. Differential Expression Pattern of the IL-2-Receptor *α*- and *β*-Chain on CD4^+^ T Cells

**Figure 3:**
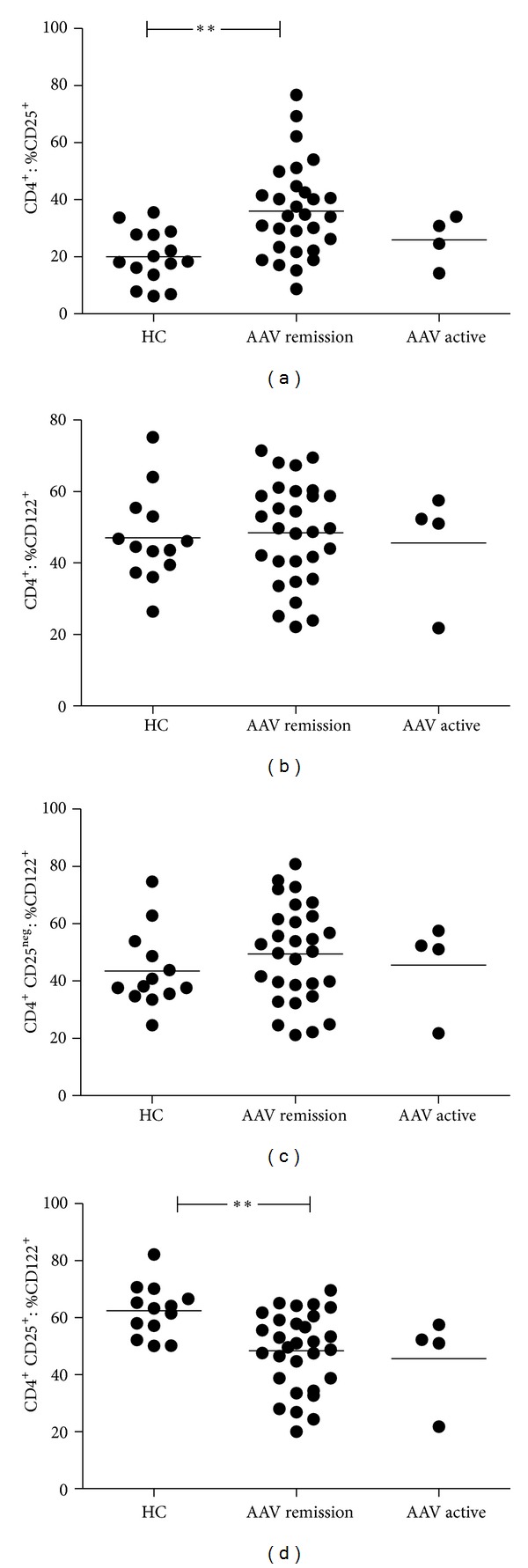
The expression of the IL-2 receptor *β*-chain is diminished on CD25^+^ CD4^+^ T cells. (a) CD25 (IL-2r *α*-chain) expression is significantly elevated on CD4^+^ T cells of AAV patients in remission as compared to HC (CD4^+^  T cells: %CD25^+^  36 ± 16% versus 20 ± 9%, *P* < 0.005). Patients with active disease were not significantly different from HC or patients in remission. (b), (c), and (d) CD122 expression is not altered on total CD4^+^  T cells and there is no difference in terms of CD122 expression on CD25^neg^  CD4^+^  T cells comparing AAV patients and HC. CD122 expression is significantly reduced on CD25^+^  CD4^+^  T cells comparing AAV in remission and HC (CD25^+^  CD4^+^  T cells: %CD122^+^  48 ± 14% versus 62 ± 9%, *P* = 0.002). Patients with active disease showed a tendency towards a lower expression of CD122 on CD25^+^  CD4^+^  T cells as compared to HC (46 ± 16% versus 62 ± 9%, *P* = 0.06). Horizontal bar indicates the mean value. **P value < 0.005.

CD4^**+**^ T cells were analysed for the expression of the IL-2r *α*-chain (CD25) and *β*-chain (CD122). As reported by several other groups before, CD25 expression on CD4^**+**^ T cells was significantly increased in patients with quiescent AAV as compared to HC (CD4^**+**^ T cells: %CD25^**+**^  36 ± 16% versus 20 ± 9%, *P* < 0.005, Figure 3(a)).

In contrast, CD122 expression on total CD4^**+**^ T cells was similar in quiescent AAV and HC (CD4^**+**^ T cells: %CD122^**+**^  48 ± 15% versus 47 ± 13%, not significant (ns), Figure 3(b)). In AAV patients in remission, CD122 expression of CD4^**+**^ T cells correlated negatively with CD25^**+**^ expression of CD4^**+**^ T cells (*r* = −0.4, *P* = 0.05). Such an association was not evident in HC (*r* = 0.02, *P* = 0.96).

**Figure 4:**
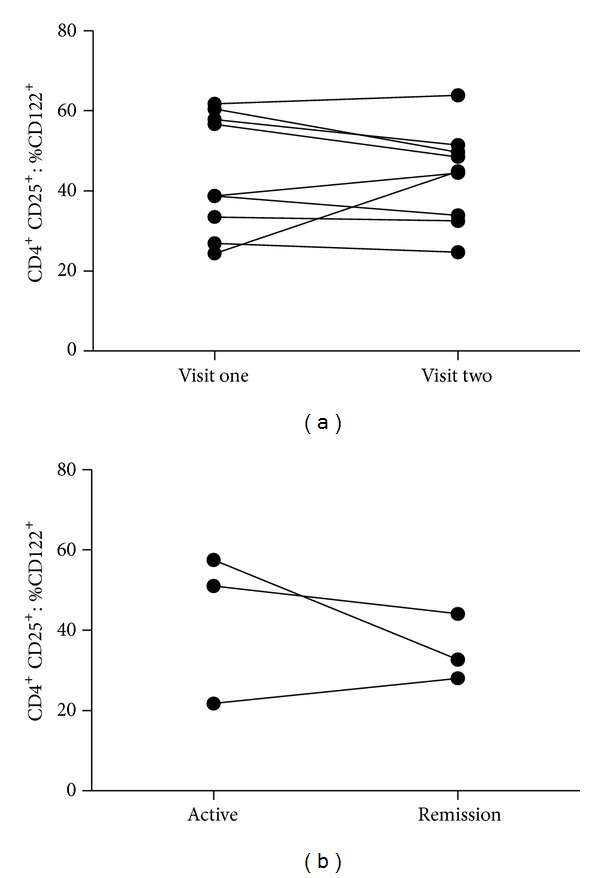
Longitudinal course of CD122 expression on CD25^+^CD4^+^ T cells. (a) Nine patients maintained remission throughout the study period and were sampled twice. The mean time between visits 1 and 2 was 8 ± 4 months. CD122 was comparable between the two visits and not significantly different. Wilcoxon signed-rank test was used to test for significance. (b) Three patients with active disease were followed longitudinally and sampled again after remission had been achieved. The expression of CD122 on CD25^+^CD4^+^ T cells remained low and did not increase. The mean time between the two visits was 10 ± 6 months.

CD25^neg^ and CD25^**+**^ CD4^**+**^ T-cell populations were then analyzed separately for the presence of CD122. Within the CD25^neg^ CD4^**+**^ T-cell subset, there was no difference in terms of CD122 expression comparing quiescent AAV and HC (CD25^neg^ CD4^**+**^  T cells: %CD122^**+**^49 ± 17% versus 44 ± 14%, ns, Figure 3(c)). However, within the CD25^**+**^  CD4^**+**^ T-cell population, CD122 expression was significantly reduced in quiescent AAV when compared to HC (CD25^**+**^ CD4^**+**^ T-cells: %CD122^**+**^  48 ± 14% versus 62 ± 9%, *P* = 0.002, Figure 3(d)). Longitudinal follow-up of nine patients with AAV who stayed in remission showed that the diminished expression of CD122 on CD25^**+**^  CD4^**+**^ T cells is stable over time (Figure 4(a)).

In patients with active disease, CD122 expression was also diminished on CD25^**+**^CD4^**+**^ T cells when compared to HC (46 ± 16% versus 62 ± 9%, *P* = 0.06, 3D). After having entered remission, CD122 expression on CD25^**+**^CD4^**+**^ T cells did not increase (Figure 4(b)).

### 3.2. CD4^+^CD25^+^FoxP3^+^ Regulatory T-Cells in AAV Largely Lack Expression of the IL-2 Receptor *β*-Chain

Since activated FoxP3^neg^ T-helper cells and FoxP3^pos^ Tregs reside both within the CD25^**+**^ CD4^**+**^ T-cell population, the transcription factor FoxP3 was used to distinguish these cell types. The fraction of Tregs was comparable in HC and AAV patients in remission (CD4^**+**^ T cells % FoxP3^**+**^CD25^**+**^  5.8 ± 3% versus 5.6 ± 2%, ns).

**Figure 5:**
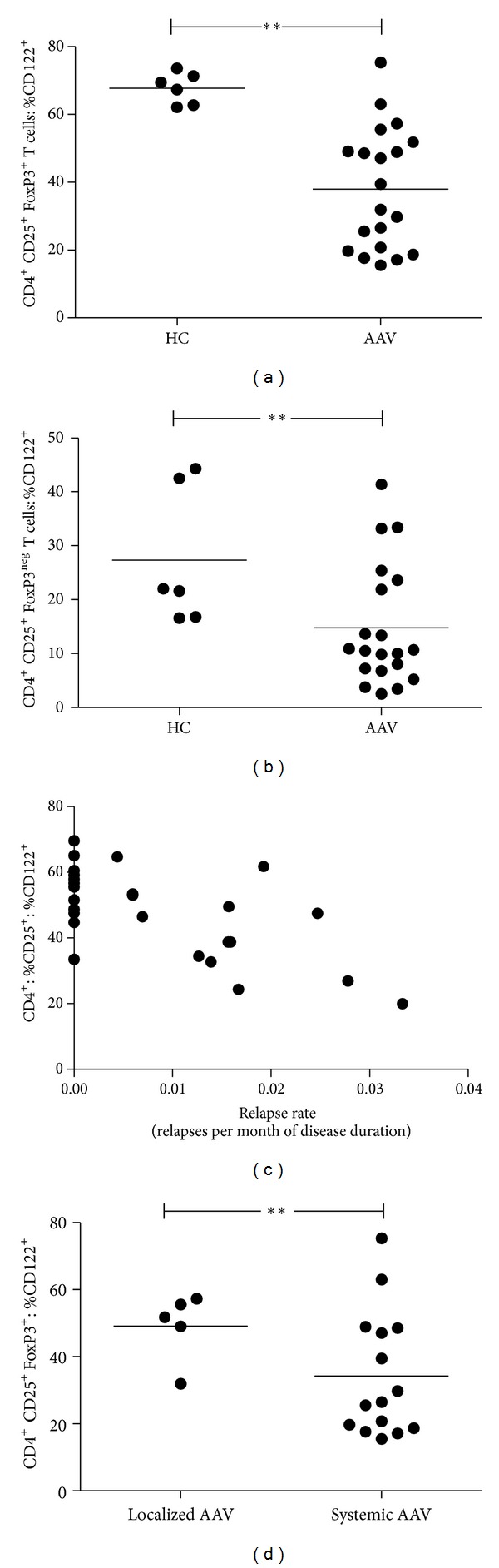
The IL-2 receptor *β*-chain is markedly reduced on Tregs in AAV and CD122 expression on CD4^+^ T cells is associated with disease parameters. (a) In contrast to HC, CD122 (IL-2r *β*-chain) is only expressed by a minority of Tregs in AAV (CD4^+^CD25^+^FoxP3^+^ Tregs: %CD122^+^  38 ± 18% versus 68 ± 5%, *P* = 0.002). (b) Likewise, CD122 expression is diminished on activated CD4^+^ T-helper cells in AAV (CD4^+^CD25^+^FoxP3^neg^ T cells: %CD122^+^  15 ± 11% versus 27 ± 13%, *P* = 0.03). (c) The relapse rate is negatively correlated with CD122 expression on CD25^+^ CD4^+^ T cells (*r* = −0.55, *P* = 0.004). The relapse rate (relapses per month) was calculated by dividing the amount of total, previous relapses by the disease duration in months. (d) Patients with systemic, renal vasculitis had lower expression of CD122 on Tregs than patients with localized, nonrenal disease (CD4^+^CD25^+^FoxP3^+^ Tregs: %CD122^+^34 ± 19% versus 49 ± 10%, *P* = 0.04). Horizontal bar indicates the mean value. *P value < 0.05 and **P value < 0.005.

Interestingly, CD122 was found only on a minority of CD4^**+**^CD25^**+**^FoxP3^**+**^ Tregs in quiescent AAV. In contrast, it was expressed by the vast majority of Tregs in HC (CD4^**+**^CD25^**+**^FoxP3^**+**^ Tregs: % CD122^**+**^  38 ± 18% versus 68 ± 5%, *P* = 0.002, Figure 5(a)). CD122 expression was also diminished on CD4^**+**^CD25^**+**^FoxP3^neg^ activated T-helper cells in AAV as compared to HC (CD4^**+**^CD25^**+**^FoxP3^neg^ T cells: %CD122^**+**^  15 ± 11% versus 27 ± 13%, *P* = 0.03, Figure 5(b)).

### 3.3. IL-2 Receptor *β*-Chain Expression on CD4^+^CD25^+^ T Cells Correlates with Relapse Rate and Systemic, Renal AAV

To further study the clinical implications of the diminished presence of CD122, a correlation analysis was performed. Patients with lower expression of CD122 had experienced more relapses as indicated by the negative correlation of CD122 expression on CD25^**+**^ CD4^**+**^ T cells and relapse rate (*r* = −0.55, *P* = 0.004, Figure 5(c)).

Interestingly, there was a negative association of serum creatinine and CD122 expression on CD25^**+**^ CD4^**+**^ T cells (*r* = −0.35, *P* = 0.06). We had to exclude that renal impairment and uremic conditions in itself have an impact on CD122 expression. Thus, CD122 expression on CD4^+^ T cells was assessed in two additional control cohorts with chronic renal impairment. CD122 expression on CD4^**+**^ T cells and CD25^**+**^ CD4^**+**^ and CD25^neg^ CD4^**+**^T cells was not different between HC and patients following nephrectomy (CD4^**+**^ T cells: %CD122^**+**^  47 ± 13% versus 43 ± 12%, ns; CD25^**+**^ CD4^**+**^ T cells: 62 ± 9% versus 54 ± 11%, ns; CD25^neg^CD4^+^ T cells: 44 ± 14% versus 41 ± 12%, ns). Furthermore, CD122 expression on CD4^**+**^ T cells and CD25^**+**^ CD4^**+**^ T cells and CD25^neg^ CD4^**+**^ T cells was not different between HC and patients with IgA-nephropathy (CD4^**+**^ T cells: %CD122^**+**^47 ± 13% versus 44 ± 11%, ns; CD4^**+**^CD25^**+**^: 62 ± 9% versus 56 ± 9%, ns; CD4^**+**^CD25^neg^: 44 ± 14% versus 41 ± 10%, ns). There was no correlation of serum creatinine levels and CD122 expression on CD25^**+**^ CD4^**+**^ T-cells in patients with IgA nephropathy (*r* = 0.4, *P* = 0.2). Thus, only in patients with AAV, lower presence of CD122 on CD4^**+**^ CD25^+^ T cells correlated with higher levels of serum creatinine, that is, worse renal function. Accordingly, patients suffering from systemic vasculitis with renal involvement had a significantly lower expression of CD122 on Tregs when compared to patients with localized, nonrenal vasculitis (CD4^**+**^CD25^**+**^FoxP3^**+**^ Tregs: %CD122^**+**^  34 ± 19% versus 49 ± 10%, *P* = 0.04, Figure 5(d)). No associations between clinical parameters and CD25 expression on CD4^**+**^ T cells of AAV patients were found.

## 4. Discussion

Our results show that the different subunits of the IL-2 receptor have an abnormal expression pattern in AAV. The *α*-chain of the IL-2r is overexpressed on CD4^**+**^ T cells in AAV, whereas the expression of the IL-2r *β*-chain CD122 is significantly diminished on activated T-helper cells and Tregs when compared to healthy controls. Interestingly, low expression of CD122 was associated with increased relapse rate, worse renal function, and renal involvement in AAV.

Our results confirm previous observations which described an overexpression of the IL-2r *α*-chain CD25 on CD4^**+**^ T cells in patients with AAV [11, 27]. CD25 as part of the high-affinity IL-2r is usually expressed constitutively by Tregs or transiently by activated T-helper cells [16, 17]. The enhanced expression of CD25 in AAV is most likely due to persistent activation of Teff as the fraction of Tregs was comparable between AAV patients and HC in line with other studies [7, 27, 28]. Chronic challenge with antigen may contribute to persistent activation [1, 2]. A similar phenomenon has been observed in other human autoimmune diseases [29, 30].

We are the first to describe the expression pattern of the IL-2r *β*-chain on CD4^**+**^ T cells in AAV. Surprisingly, the expression of the IL-2r *β*-chain CD122 was sharply diminished on CD25^**+**^ CD4^**+**^ T cells, activated T-helper cells and Tregs in AAV. Under physiological conditions, activated T-helper cells and Tregs should express the heterotrimeric high-affinity IL-2r which consists of all three subunits [15, 16]. Accordingly, in HC, more than half of the CD25^**+**^ CD4^**+**^ T cells and about 70% of the Tregs coexpressed the IL-2r *β*-chain CD122 along with the *α*-chain CD25. CD122 is indispensable for proper function of the IL-2r as it is critically involved in signal transduction with its cytoplasmic domain [16]. Therefore, CD25^**+**^ CD4^**+**^ T cells and Tregs lacking CD122 may show decreased responsiveness to IL-2. IL-2 is considered as a Treg “growth factor” promoting Treg differentiation and survival [15]. Deprivation of IL-2 due to a dysfunctional receptor may lead to diminished Treg development, Treg apoptosis, reduced suppressive function, or even loss of lineage commitment with permanent downregulation of FoxP3 [15, 16, 31, 32]. Indeed, it has been demonstrated before that Tregs are functionally impaired in AAV and fail to suppress effector T cells [13, 14, 28]. Moreover, it has recently been reported that decreased IL-10 production is associated with a higher risk for relapse [33]. Reduced sensitivity of Tregs to IL-2 may be one of the mechanisms which contributes to Treg dysfunction and decreased IL-10 production [34]. In line with this, defective IL-2 signaling in type 1 diabetic patients results in loss of the essential transcription factor FoxP3 in Tregs leading to dysfunctional suppression [34, 35].

Interestingly, CD122 is also expressed on a regulatory subset of CD8^**+**^ T cells [36]. These CD8^**+**^ Tregs promote immune tolerance, prevent autoimmunity, and inhibit effector CD8^**+**^ T cells which lack CD122 [36]. It is tempting to speculate that the alterations of the *β*-chain expression in AAV may not only affect CD4^**+**^ T cells but also CD8^**+**^ T cells. However, the focus of our study was CD4^**+**^ T cells and thus the impact on CD8^**+**^CD122^**+**^ T cells in AAV remains unclear. The reason for the diminished presence of CD122 remains elusive. A genetic basis for AAV was recently demonstrated by a large genome wide association study [37]. Although not proven in the aforementioned GWAS, genetic alterations may play a role in the aberrant expression pattern of the IL-2r in AAV. A previous study by Carr et al. reported an association of a specific gene polymorphism coding for the *α*-chain of the IL-2r and AAV [38]. However, an association of AAV and a specific polymorphism of the gene coding for the *β*-chain of the IL-2r has not been reported so far. We cannot exclude that immunosuppressive treatment influenced the expression of CD122 because the majority of patients were treated with immunosuppressants in our study. Due to the relapsing nature of AAV, patients have to be treated with immunosuppression even if remission is achieved. Longitudinal data covering start of treatment and cessation of immunosuppressants will help to clarify the impact of medication on CD122 expression.

Lastly, CD122 expression on CD25^**+**^CD4^**+**^ T cells was stable over time during remission and correlated with the relapse rate. This indicates that the course of the disease is related to CD122 expression. It is not clear from our study if low CD122 expression is the cause or consequence of an increased relapse rate. The value of CD122 as a biomarker for risk of relapse remains to be studied in a prospective study approach.

## 5. Conclusion

In conclusion, there is an aberrant expression pattern of the IL-2r in AAV. Whereas the *α*-chain is overexpressed, the expression of the *β*-chain is sharply diminished on activated T-helper cells and Treg. The reduced expression of the *β*-chain CD122 was associated with a less favorable disease course. Given the critical role of the IL-2/IL-2r pathway in immune homeostasis, one may speculate that the abnormal expression pattern of the IL-2r may be a contributing factor in the pathogenesis of AAV.

## Figures and Tables

**Table 1:** Clinical characteristics of the patient cohort in remission.

Cohort characteristics of patients in remission	Systemic disease	Localized disease
*n*	22	8
Mean age (years)	62 ± 13	48 ± 11
Gender (female/male)	13/9	1/7
PR3/MPO	21/1	8/0
Mean disease duration (months)	115 ± 80	68 ± 69
Maintenance treatment *y*/*n*	22/0	8/0
Relapse rate (previous relapses per month of disease duration)	0.01 ± 0.01	0.005 ± 0.007
Mean glomerular filtration rate (as calculated by MDRD, mL/min)	41 ± 17	60 ± 0

## References

[1] Wilde B, van Paassen P, Witzke O, Tervaert JWC (2011). New pathophysiological insights and treatment of ANCA-associated vasculitis. *Kidney International*.

[2] Wilde B, Thewissen M, Damoiseaux J, van Paassen P, Witzke O, Tervaert JWC (2010). T cells in ANCA-associated vasculitis: what can we learn from lesional versus circulating T cells?. *Arthritis Research and Therapy*.

[3] Brouwer E, Cohen Tervaert JW, Horst G (1991). Predominance of IgG1 and IgG4 subclasses of anti-neutrophil cytoplasmic autoantibodies (ANCA) in patients with Wegener’s granulomatosis and clinically related disorders. *Clinical and Experimental Immunology*.

[4] Wilde B, van Paassen P, Damoiseaux J (2009). Dendritic cells in renal biopsies of patients with ANCA-associated vasculitis. *Nephrology Dialysis Transplantation*.

[5] Lamprecht P, Moosig F, Csernok E (2001). CD28 negative T cells are enriched in granulomatous lesions of the respiratory tract in Wegener’s granulomatosis. *Thorax*.

[6] Lamprecht P, Csernok E, Gross WL (2006). Effector memory T cells as driving force of granuloma formation and autoimmunity in Wegener’s granulomatosis. *Journal of Internal Medicine*.

[7] Wilde B, Thewissen M, Damoiseaux J (2012). Th17 expansion in granulomatosis with polyangiitis (Wegener's): the role of disease activity, immune regulation and therapy. *Arthritis Research & Therapy*.

[8] Wilde B, Hua F, Dolff S (2012). Aberrant expression of the negative costimulator PD-1 on T cells in granulomatosis with polyangiitis. *Rheumatology*.

[9] Wilde B, Dolff S, Cai X (2009). CD4+CD25+ T-cell populations expressing CD134 and GITR are associated with disease activity in patients with Wegener’s granulomatosis. *Nephrology Dialysis Transplantation*.

[10] Stegeman CA, Cohen Tervaert JW, Huitema MG, Kallenberg CGM (1993). Serum markers of T cell activation in relapses of Wegener’s granulomatosis. *Clinical and Experimental Immunology*.

[11] Popa ER, Stegeman CA, Bos NA, Kallenberg CGM, Tervaert JWC (1999). Differential B- and T-cell activation in Wegener’s granulomatosis. *Journal of Allergy and Clinical Immunology*.

[12] Marinaki S, Kälsch A-I, Grimminger P (2006). Persistent T-cell activation and clinical correlations in patients with ANCA-associated systemic vasculitis. *Nephrology Dialysis Transplantation*.

[13] Abdulahad WH, Stegeman CA, van der Geld YM, Doornbos-van der Meer B, Limburg PC, Kallenberg CGM (2007). Functional defect of circulating regulatory CD4+ T cells in patients with Wegener’s granulomatosis in remission. *Arthritis and Rheumatism*.

[14] Free ME, Bunch DO, McGregor J (2013). ANCA-associated vasculitis patients have defective Treg function exacerbated by presence of a suppression-resistant effector population. *Arthritis & Rheumatism*.

[15] Malek TR, Bayer AL (2004). Tolerance, not immunity, crucially depends on IL-2. *Nature Reviews Immunology*.

[16] Malek TR (2008). The Biology of Interleukin-2. *Annual Review of Immunology*.

[17] Létourneau S, Krieg C, Pantaleo G, Boyman O (2009). IL-2- and CD25-dependent immunoregulatory mechanisms in the homeostasis of T-cell subsets. *Journal of Allergy and Clinical Immunology*.

[18] Rochman Y, Spolski R, Leonard WJ (2009). New insights into the regulation of T cells by *γ*c family cytokines. *Nature Reviews Immunology*.

[19] Burchill MA, Yang J, Vang KB, Farrar MA (2007). Interleukin-2 receptor signaling in regulatory T cell development and homeostasis. *Immunology Letters*.

[20] Soper DM, Kasprowicz DJ, Ziegler SF (2007). IL-2R*β* links IL-2R signaling with Foxp3 expression. *European Journal of Immunology*.

[21] Liao W, Lin J-X, Leonard WJ (2011). IL-2 family cytokines: new insights into the complex roles of IL-2 as a broad regulator of T helper cell differentiation. *Current Opinion in Immunology*.

[22] Suzuki H, Kundig TM, Furlonger C (1995). Deregulated T cell activation and autoimmunity in mice lacking interleukin-2 receptor *β*. *Science*.

[23] Hellmich B, Flossmann O, Gross WL (2007). EULAR recommendations for conducting clinical studies and/or clinical trials in systemic vasculitis: focus on anti-neutrophil cytoplasm antibody-associated vasculitis. *Annals of the Rheumatic Diseases*.

[24] Leavitt RY, Fauci AS, Bloch DA (1990). The American College of Rheumatology 1990 criteria for the classification of Wegener’s granulomatosis. *Arthritis and Rheumatism*.

[25] Jennette JC, Falk RJ, Andrassy K (1994). Nomenclature of systemic vasculitides: proposal of an international consensus conference. *Arthritis and Rheumatism*.

[26] Jennette JC, Falk RJ, Bacon PA (2013). 2012 revised international Chapel Hill consensus conference nomenclature of vasculitides. *Arthritis & Rheumatism*.

[27] Marinaki S, Neumann I, Kälsch A-I (2005). Abnormalities of CD4+ T cell subpopulations in ANCA-associated vasculitis. *Clinical and Experimental Immunology*.

[28] Morgan MD, Day CJ, Piper KP (2010). Patients with Wegener’s granulomatosis demonstrate a relative deficiency and functional impairment of T-regulatory cells. *Immunology*.

[29] Han GM, O’Neil-Andersen NJ, Zurier RB, Lawrence DA (2008). CD4+CD25high T cell numbers are enriched in the peripheral blood of patients with rheumatoid arthritis. *Cellular Immunology*.

[30] Mesquita D, de Melo Cruvinel W, Araujo JAP (2011). Systemic lupus erythematosus exhibits a dynamic and continuum spectrum of effector/regulatory T cells. *Scandinavian Journal of Rheumatology*.

[31] Setoguchi R, Hori S, Takahashi T, Sakaguchi S (2005). Homeostatic maintenance of natural Foxp3+ CD25+ CD4+ regulatory T cells by interleukin (IL)-2 and induction of autoimmune disease by IL-2 neutralization. *Journal of Experimental Medicine*.

[32] Cheng G, Yu A, Dee MJ, Malek : TR (2013). IL-2R signaling is essential for functional maturation of regulatory T cells during thymic development. *The Journal of Immunology*.

[33] Hruskova Z, Casian AL, Konopasek P (2013). Long-term outcome of severe alveolar haemorrhage in ANCA-associated vasculitis: a retrospective cohort study. *Scandinavian Journal of Rheumatology*.

[34] Garg G, Tyler JR, Yang JHM (2012). Type 1 diabetes-associated IL2RA variation lowers IL-2 signaling and contributes to diminished CD4+CD25+ regulatory T cell function. *Journal of Immunology*.

[35] Long SA, Cerosaletti K, Bollyky PL (2010). Defects in IL-2R signaling contribute to diminished maintenance of FOXP3 expression in CD4+CD25+ regulatory T-cells of type 1 diabetic subjects. *Diabetes*.

[36] Rifa’i M, Kawamoto Y, Nakashima I, Suzuki H (2004). Essential roles of CD8+CD122+ regulatory T cells in the maintenance of T cell homeostasis. *Journal of Experimental Medicine*.

[37] Lyons PA, Rayner TF, Trivedi S (2012). Genetically distinct subsets within ANCA-associated vasculitis. *The New England Journal of Medicine*.

[38] Carr EJ, Clatworthy MR, Lowe CE (2009). Contrasting genetic association of IL2RA with SLE and ANCA—associated vasculitis. *BMC Medical Genetics*.

